# Fermented milk protein consumption improves exercise performance and total body mass in prepubertal children: a randomized double-blind, placebo-controlled pilot trial

**DOI:** 10.3389/fnut.2026.1755943

**Published:** 2026-03-18

**Authors:** Atsushi Kanda, Hironaga Ito, Ryosuke Takahashi, Yuki Urushizawa, Kei Tsukioka, Chiaki Sanbongi

**Affiliations:** 1 R & D Division, Meiji Co., Ltd., Tokyo, Japan; 2 Euphoria Co., Ltd., Tokyo, Japan

**Keywords:** development, exercise, fermented milk, growth, gut microbiota, milk protein

## Abstract

**Introduction:**

Milk proteins are highly digestible and contain essential amino acids. Fermentation by lactic acid bacteria can modify protein characteristics and may influence digestion and downstream responses. Direct human comparisons of fermented versus non-fermented milk protein in prepubertal children are lacking. In this pilot randomized, double-blind, placebo-controlled trial, we examined whether an 8-week fermented milk protein beverage affects exercise performance, body composition, and gut microbiota in prepubertal boys.

**Methods:**

A randomized, double-blind, parallel-group controlled trial was conducted in 44 healthy boys aged 10–12 years who regularly played soccer. Participants were randomly assigned to one of three groups: fermented milk protein beverage (FM; 93 kcal, 12 g protein/200 mL), milk protein beverage (MP; 93 kcal, 12 g protein/200 mL), or placebo beverage (PL; 93 kcal, 0 g protein/200 mL) group. Each group was instructed to consume the assigned beverage daily for 8 weeks. The prespecified primary outcome was the change in 10-m sprint time; and secondary outcomes included 20-m sprint time, countermovement jump, standing long jump, Yo-Yo test, total body mass, lean body mass (LBM), and exploratory gut microbiota analyses.

**Results:**

Compared with the PL group, both the FM and MP groups showed significant improvements in 10-m sprint time based on pre- to post-intervention changes (0.015 ± 0.013, −0.024 ± 0.013, and −0.045 ± 0.015 s for PL, FM, and MP, respectively). The FM group also exhibited a significantly greater increase in total body mass than the PL group (0.85 ± 0.19 kg vs. 0.28 ± 0.19 kg, respectively). No significant between-group differences were observed for LBM or other performance outcomes. Exploratory microbiota analyses indicated a within-group increase in *Bacteroides massiliensis* in FM and associative (non-causal) correlations with phenotype changes.

**Conclusion:**

In this pilot study, daily fermented milk protein intake was associated with modest improvements in short-distance sprint performance and increased total body mass vs. placebo, while superiority over non-fermented milk protein was not consistently demonstrated. Larger trials are needed to confirm these findings and clarify mechanisms.

**Clinical trial registration:**

https://rctportal.mhlw.go.jp/en/detail?trial_id=UMIN000055618, UMIN-CTR UMIN000055618.

## Graphical abstract

**Figure:**
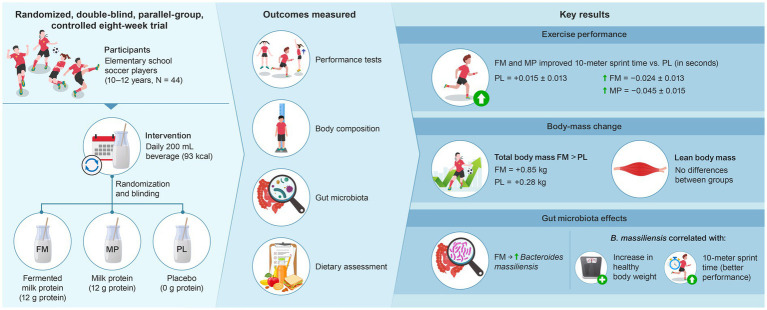

## 1 Introduction

Milk is a nutritious protein source with an excellent balance of essential amino acids. Although several methods exist for measuring protein nutritional quality, milk protein has been shown to rank highly regardless of the method used, including the recently established protein quality standard, the Digestible Indispensable Amino Acid Score (DIAAS) (1–3). Milk protein is rich in branched-chain amino acids (BCAAs), which are crucial for muscle formation and highly effective in enhancing muscle synthesis (4). Consequently, it is used as a supplement by athletes worldwide. Milk proteins are mainly composed of two types of proteins, whey protein and casein, at an approximate ratio of 2:8, respectively. Both whey protein and casein possess high nutritional value; however, the primary difference lies in their rates of digestion and absorption. Whey proteins are rapidly absorbed by the body after ingestion (5, 6). In contrast, casein is slowly digested and absorbed because its aggregation under acidic conditions causes it to coagulate in the stomach and be gradually digested and absorbed by the intestinal tract (5, 6). Whey protein is particularly effective in enhancing muscle synthesis because of its rapid absorption and ability to quickly elevate blood amino acid concentrations (4, 5).

Fermented milk is primarily produced by the fermentation of milk by lactic acid bacteria. It is widely consumed globally, mainly in the form of yogurt and cheese, and has a long history. Fermented milk contains whey protein and casein in roughly the same proportion as milk, although some of these proteins are partially hydrolyzed during fermentation. Furthermore, casein in fermented milk does not coagulate or precipitate in the stomach as rapidly as when milk and acid meet. These factors suggest a potential difference in protein absorption between milk and fermented milk (7). Using the total amino acid (TAA) concentration in rat portal blood as an index of protein digestion and absorption, multiple studies have demonstrated that fermented milk results in higher blood TAA concentrations than milk (8, 9). Hence, fermented milk is more digestible and is readily absorbed. Furthermore, recent research regarding the utilization of protein by skeletal muscles following fermented milk ingestion indicates that fermented milk enhances the rate of skeletal muscle synthesis more effectively than milk (8, 9). However, studies in human participants are limited, and further investigations are necessary (7).

The prepubertal period is a phase of rapid growth and development that requires adequate nutrients for growth (10, 11). Therefore, prepubertal children engaged in sports activities must increase their nutrient intake sufficiently while compensating for increased energy expenditure (12). Childhood and adolescence are critical developmental periods during which protein intake plays an extremely important role in musculoskeletal health (10). Proteins promote muscle synthesis and, over the long term, maintain and increase lean body mass (LBM). Increased dietary protein intake has been shown to be significantly and positively associated with both Appendicular Lean Mass Index (ALMI) and grip strength, which are indices of musculoskeletal health in children and adolescents (10). A large-scale cohort study targeting prepubertal girls in the United States demonstrated that intakes of dairy milk, fermented milk, and milk protein were positively associated with subsequent height gain, peak height velocity (PHV), and final adult height (13). Studies on growing animals have shown that diets containing fermented milk result in a greater increase in body weight per unit protein intake than those containing unfermented milk (14, 15). These results suggest that milk protein intake is important for children’s growth and that fermented milk protein consumption, in particular, may be effective; however, no prior research has compared the effects of intervention with fermented milk versus unfermented milk, specifically in prepubertal children.

There is growing interest in the close relationship between the state of skeletal muscles and the gut microbiota, often referred to as the ‘gut-muscle axis’ (7). The gut microbiota is involved in regulating human metabolism, energy balance, fat deposition, liver function, and skeletal muscle function, suggesting that modulating its composition is crucial for maintaining health (16). Fermented milk consumption has been suggested to beneficially modulate the gut microbiota and potentially reduce the risk of overweight and obesity in adults (17). However, no prior research has investigated the effects of fermented milk consumption on the gut microbiota, total body mass, and body composition in prepubertal children.

Milk proteins—primarily whey protein and casein—are highly rated across protein quality metrics and widely used in sports settings. Fermentation by lactic acid bacteria partially hydrolyzes milk proteins, alters gastric behavior, and may modify digestion and absorption kinetics. Building on experimental work suggesting enhanced postprandial amino-acid availability and muscle protein synthesis (MPS) after fermented milk intake, we hypothesized that a fermented milk protein beverage could be beneficial for growing children engaged in regular sports.

Importantly, to the best of our knowledge, no prior human study has directly compared fermented versus non-fermented milk protein in prepubertal young athletes. Therefore, we designed a randomized, double-blind, placebo-controlled pilot trial to evaluate the effects of 8 weeks of fermented milk protein consumption on exercise performance, anthropometrics, and gut microbiota, compared with both a non-fermented milk protein beverage and an isocaloric placebo.

Through this study, we aimed to generate hypothesis-forming evidence while acknowledging that any mechanistic interpretations would remain speculative, particularly for microbiota–phenotype associations.

## 2 Methods

### 2.1 Study design

This was a double-blind, parallel-group, randomized controlled trial. Forty-four elementary school soccer players were recruited for the study after they provided consent for participation. The inclusion criteria included: male sex, 10–12 years of age, and regular soccer participation at least once a week. The exclusion criteria included: individuals with physical, intellectual, or mental disabilities that would make it difficult to perform physical fitness tests in the same way as healthy individuals; individuals with a medical condition, such as heart disease, that medically prohibited full participation in sports and exercise; and individuals with allergies, lactose intolerance, or other conditions that could worsen their health by consuming the study beverage. An explanatory document detailing the study was distributed to the participants and their parents, and consent for participation was obtained from the parents. After confirming participation in the study, the participants were randomly allocated to one of the three supplemented groups. All participants then started an 8 week supplement intervention. Exercise performance and body composition were assessed before and after the intervention. The trial was conducted by Euphoria Co. Ltd. (Tokyo, Japan) between September and December 2024. Data were analyzed between January and March 2025. The present study was considered a pilot study because no previous studies have investigated the effects of yogurt protein supplementation on exercise performance and body composition in prepubertal children. A target of 15 participants per group was set to allow for potential attrition and ensure a final sample size of more than 12 participants per group (18).

### 2.2 Ethics approval

This study was registered in the UMIN Clinical Trials Registry (registration number: UMIN000055618). The procedures used in this study were approved by the Institutional Review Board of Euphoria Co., Ltd. (approval number: eu-00010). The study was conducted in accordance with the ethical principles of the Declaration of Helsinki and the Ethical Guidelines for Medical and Health Research Involving Human Subjects (Ministry of Health, Labor and Welfare, Japan).

### 2.3 Randomization and blinding

After obtaining informed consent, pre-intervention measurements were performed before the participants began consuming the test beverages. Within 3 weeks of the pre-intervention measurements, participants were stratified by their team affiliation and growth phase and then block-randomly assigned to one of three groups: the placebo (PL) beverage group, acidified milk protein (MP) beverage group, or fermented milk protein (FM) beverage group. Allocation was done by an individual who was not involved in the planning, enrollment, evaluation, intervention, or analysis of the study. The participants, investigators, and staff members involved in the trial were blinded to group allocation. The growth phase was determined based on the peak height velocity (PHV). The age difference from the peak height velocity (PHVA) was calculated from the chronological age, height, weight, sitting height, and leg length (height minus sitting height) (19). The maturity stages were then categorized as pre-PHV if the age difference was less than −1, circa-PHV if it was between −1 and +1 (inclusive), and post-PHV if it was greater than +1 (20). Post-intervention measurements were conducted within 1 week of the final day of beverage consumption. The randomization code was opened after the study data were checked, collated, and finalized.

### 2.4 Supplementation protocol

After randomization, the participants consumed their assigned test beverages for 8 weeks. Beverages were consumed daily in the morning. To make the test beverages indistinguishable from each other, they were packaged in plain brick-type cartons with uniform white straws. The FM beverage was prepared from whey protein concentrate, skim milk, maltodextrin, soybean polysaccharide, pectin, lactic acid, emulsifier, sucralose, and food flavors, and fermented with the culture LB81, which contains *Lactobacillus delbrueckii ssp. bulgaricus 2038* and *Streptococcus thermophilus 1,131* (21). The FM beverage contained 9.3 g of carbohydrate, 12.1 g of protein, and 1.1 g of fat, providing 93 kcal of energy per carton. The FM beverage contained a total of 2.6 × 10^11^ CFU of lactic acid bacteria in heat-inanimate form. The MP beverage was made from whey protein concentrate, skim milk, soybean polysaccharide, pectin, lactic acids, emulsifier, sucralose, and food flavors, and contained 9.2 g of carbohydrate, 12.1 g of protein, and 1.1 g of fat, providing 93 kcal of energy per carton. The PL beverage was made from maltodextrin, soybean polysaccharide, pectin, lactic acid, emulsifier, sucralose, and food flavors, and contained 23.3 g of carbohydrate, 0.1 g of protein, and 0.1 g of fat, providing 93 kcal of energy per carton. The test drinks had an identical pH (4.0). Test beverage consumption and soccer practice time were monitored by having the participants complete a designated log sheet or online form.

### 2.5 Measurements

Pre- and post-intervention measurements were performed in a gymnasium or covered futsal court between 6:00 p.m. and 8:00 p.m. Upon arrival, the research staff confirmed the participants’ health status and absence of injuries before measuring their height, sitting height, and body composition. Following these measurements, a warm-up including a 5-min run and dynamic stretching was conducted. Subsequently, the participants performed a 20-m sprint, standing long jump, countermovement jump (CMJ), rebound jump, and Yo-Yo intermittent recovery test 1 (YYIRT1), in that order. A single practice trial was allowed for each measurement before the actual test, except for the YYIRT1.

#### 2.5.1 20 m linear sprint

The time required for a linear sprint was measured to assess sprinting ability. Photocell timing gates (WITTY, Microgate Srl; Bolzano, Italy; 0.001-s accuracy) were set up at the start line, 10-m mark, and 20-m mark to measure the sprint times at 10 m and the final time at 20 m. The starting line was placed 50 cm behind the timing gate to ensure that the participant’s torso did not interrupt the infrared beam at the beginning of the run. A sprint start position was used. Two trials were performed, and the 10 and 20 m sprint times corresponding to the fastest 20 m trial were used for the analysis.

#### 2.5.2 CMJ

A CMJ was performed to evaluate vertical lower-body power, and the jump height was measured. The participants stood shoulder-width apart with their feet and hands on their hips. They performed a quick squat to initiate countermovement and then jumped to the maximum height. The squat depth was selected for each participant. Jump height was the outcome variable in this study. Three trials were performed and the maximum value was used for the analysis. Measurements were taken using a jump mat (S-CADE, Tokyo, Japan) and the flight time method.

#### 2.5.3 Standing jump

A standing jump was performed to evaluate horizontal power output. The participants jumped forward from the take-off line with maximum effort. The jump distance was measured from the take-off line to the heel closest to the line upon landing using a tape measure. Two trials were performed and the maximum value was used for the analysis.

#### 2.5.4 YYIRT1

To evaluate intermittent endurance, the YYIRT1 was conducted according to the guidelines of Krustrup et al. (22). The participants repeatedly ran 20-m shuttles in time using audio signals. The interval between the signals decreased as the test progressed, thereby increasing intensity. The total number of shuttles completed and the total distance covered were used as evaluation metrics. The test was terminated when the participant failed to reach the finish line on time with the audio signal for a second time.

#### 2.5.5 Body composition

A direct segmental multifrequency (5, 50, and 250 kHz) bioelectrical impedance analysis (DSM-BIA) device with an 8-point tactile electrode system (InBody 430, InBody Japan, Tokyo, Japan) was used to measure body weight and LBM. Body composition assessments were performed between 6:00 p.m. and 7:00 p.m. before the physical performance tests.

#### 2.5.6 Gut microbiota analysis

Fecal DNA extraction and gut microbiota analysis were performed by Proumed Co. Ltd. (Tokyo, Japan). Briefly, sequencing libraries targeting the 16S internal transcribed spacer (ITS)–23S rRNA regions were prepared, and sequencing was performed on a GridION platform (Oxford Nanopore Technologies, Oxford, UK). Taxonomic assignment was performed using the MIrROR (Microbial Identification using rRNA Operon Region) pipeline. Alpha diversity analysis was performed using the PhyloSeq package. Differential abundance analysis of bacterial taxa was performed using the DESeq2 package to assess the differences between groups (inter-group comparison) and values between the pre-intervention (baseline) and post-intervention time points within each group (intra-group comparison). The results were adjusted for multiple testing using the Benjamini–Hochberg false discovery rate (FDR) correction.

#### 2.5.7 Dietary analysis

Before and at the end of the intervention period, participants completed a brief diet history questionnaire for Japanese children and adolescents (BDHQ15y) to assess their habitual dietary intake during the previous month (23). Participants completed the questionnaire with the assistance of their parents. After completion, the questionnaires were collected and sent to an external institution (Gender Medical Research Co., Ltd., Tokyo, Japan) for dietary intake assessment.

### 2.6 Statistical analysis

The primary endpoint was the change in 10-m sprint time. Secondary endpoints included changes in 20-m sprint time, CMJ height, standing long jump distance, YYIRT1 performance, total body mass, and LBM. Microbiota outcomes and all correlation analyses were considered exploratory.

Statistical analysis was performed with the comparison of changes from baseline as the primary analysis and the comparison of measured values as the secondary analysis. A linear mixed-effects model (LMM) was used, with the change from baseline for each outcome variable as the dependent variable. Group, time, baseline value for each outcome variable, and group × time interaction were specified as fixed effects, whereas participant ID was specified as a random effect. Tests were conducted on the estimated marginal means obtained from the model. The statistical significance of the inter-group differences was tested using the emmeans package. The significance level was set at less than 5% (*p* < 0.05), and a *p*-value of less than 10% (*p* < 0.1) was interpreted as a trend. A closed testing procedure was used to control for multiplicity in the inter-group comparisons of changes from baseline. Comparisons were conducted in a predetermined order and stopped when the *p*-value became non-significant. The order of comparisons was as follows: PL group vs. FM group, PL group vs. MP group, and FM group vs. MP group. Because the LMM assumes normally distributed residuals, model-based results are reported as estimated means ± estimated standard errors (SEM).

Before comparing the measured values, the normality of each outcome variable was confirmed using the Shapiro–Wilk test. Because normality was not observed, non-parametric tests were used for both inter- and intra-group comparisons. Because inter-group comparisons of measured values were performed as secondary analyses, multiple comparison corrections were not applied. Intra-group comparisons of measured values were performed using the Wilcoxon signed-rank test. The significance level was set at less than 5% (*p* < 0.05), and a *p*-value of less than 10% (*p* < 0.1) was interpreted as a trend. These non-parametric results are reported as medians [interquartile ranges, IQRs].

To investigate the association between the gut microbiota and phenotypic traits, Spearman’s rank correlation analysis was performed. Specifically, the analysis was limited to examining the correlations between bacterial taxa and phenotypic parameters that exhibited statistically significant differences (*p* < 0.05) among the comparison groups. The Spearman’s rank correlation coefficient (|*ρ*|) ranges from −1 to 1, where the absolute value (|ρ|) indicates the strength of the association: a value close to 1 (e.g., ρ = 1 or ρ = −1) indicates a strong association (perfect monotonic association), while a value close to 0 indicates a weak association (little or no monotonic association). The sign of the coefficient determines the direction of the monotonic relationship (positive or negative), and the statistical significance of the correlation is determined based on the corresponding *p*-values.

Post-hoc subgroup analyses by energy balance were exploratory and hypothesis-generating. Energy balance was calculated using Equation 1. Energy intake was estimated from the responses to the BDHQ obtained during pre-intervention measurements. Energy requirements were estimated using Equation 1, which is based on the Dietary Reference Intakes for Japanese 2025 (24), considering basal metabolic rate, physical activity level (moderate), and energy storage. The group with an energy balance of 0 or greater was defined as the energy-high group, and the group with an energy balance of less than 0 was defined as the energy-low group.


$$\begin{array}{l} \text{Energy Balance}=\text{Energy Intake}-\text{Estimated Energy}\;\\ \text{Requirement} \end{array}$$ (1)

$$\begin{array}{l} \text{Estimated}\;\\ \text{Energy Requirement}=[\begin{array}{l} (\begin{array}{l} \text{Basal Metabolic}\;\\ \text{Rate}\;\mathrm{per}\;\mathrm{kg}\times \\ \text{Body Weight} \end{array})+\\ \text{Energy Storage} \end{array}]\times \text{Physical Activity}\;\\ \text{Level}\;(\text{Moderate}) \end{array}$$ (2)

For the exploratory analysis, only the change from baseline between the FM and PL groups was compared. Cohen’s effect size *d* was calculated to evaluate the practical magnitude of group differences. The calculated *d*-values were interpreted as small (*d* = 0.2), medium (*d* = 0.5), or large (*d* = 0.8).

Reporting conventions: throughout the manuscript, model-based change analyses are presented as estimated means ± SEM, whereas descriptive values and non-parametric comparisons are presented as medians [IQRs] for clarity and consistency with the respective statistical frameworks.

Statistical analyses were conducted using R software version 4.4.2 (R Software for Statistical Computing, Vienna, Austria). The R packages used for each test were as follows: base R for the Mann–Whitney U test; effsize for Cohen’s *d*; and lme4, lmerTest, and emmeans for the LMM.

## 3 Results

### 3.1 Participants

**Figure 1:**
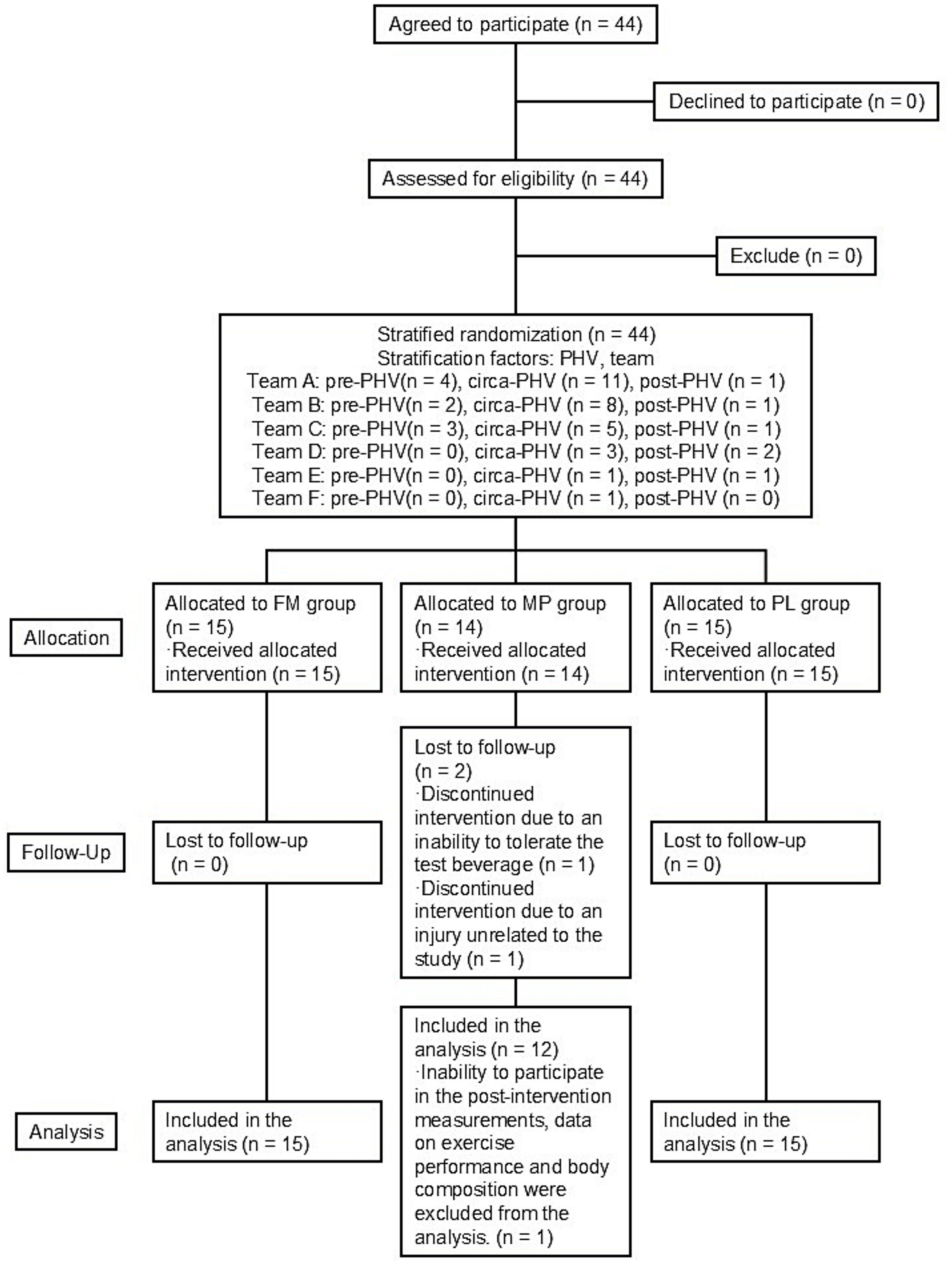
CONSORT flowchart for the intervention study.

The flow of participants through the protocol is shown in Figure 1. All participants were included in the study and randomly allocated to the FM (*n* = 15), MP (*n* = 14), or PL (*n* = 15) groups. All the participants received the allocated interventions; however, one participant discontinued the intervention due to an inability to tolerate the test beverage (MP), and one participant discontinued the intervention because of an injury unrelated to the study. Therefore, 42 participants completed the 8-week intervention. Based on the Full Analysis Set principle, 42 participants (FM = 15, MP = 12, PL = 15) who began the allocated intervention were included in the analysis. However, one participant from the MP group was unable to participate in the post-intervention measurements; hence, their data on exercise performance and body composition were excluded from the analysis (FM = 15, MP = 11, PL = 15). The representative characteristics of the participants measured at baseline are shown in Table 1. There were no significant differences in any of the study outcomes between the three groups at baseline. Analysis of dietary data revealed that energy, protein, fat, and carbohydrate intakes did not differ between or within the groups at baseline or during the study period (Table 2).

**Table 1:** Baseline participant characteristics.

Variable	FM	MP	PL
	(*n* = 15)	(*n* = 12)	(*n* = 15)
Age (years)	11.3 ± 0.2	11.2 ± 0.2	11.0 ± 0.1
Height (cm)	141.6 ± 2.2	142.7 ± 2.3	140.1 ± 2.2
Body mass (kg)	35.1 ± 2.1	36.3 ± 3.0	34.6 ± 1.5
Lean body mass (kg)	29.6 ± 1.3	29.9 ± 1.4	28.5 ± 1.0

FM, fermented milk protein; MP, milk protein; PL, placebo.

**Table 2:** Daily dietary intake (except for test drinks) pre- and post-intervention.

Variable	FM (*n* = 15)		MP (*n* = 12)		PL (*n* = 15)	
	Pre	Post	Pre	Post	Pre	Post
Energy (kcal/kg BW/day)	69.3 (58.6, 83.3)	65.9 (55.9, 79.9)	62.1 (55.8, 77.5)	63.9 (50.9, 67.9)	65.0 (46.5, 90.8)	66.4 (51.7, 85.8)
Protein (g/kg BW/day)	2.5 (2.0, 3.0)	2.4 (1.9, 3.0)	2.3 (1.9, 2.8)	2.1 (2.0, 2.7)	2.4 (1.8, 2.6)	2.4 (2.0, 2.7)
Fat (g/kg BW/day)	2.3 (2.0, 2.7)	2.4 (2.0, 2.8)	2.2 (1.8, 2.9)	2.1 (1.9, 2.3)	2.0 (1.4, 2.9)	2.0 (1.7, 3.0)
Carbohydrate (g/kg BW/day)	9.9 (7.9, 11.7)	8.4 (6.9, 10.3)	8.1 (7.7, 9.7)	8.5 (6.5, 9.3)	9.3 (6.5, 12.7)	8.3 (6.3, 13.0)

Values are presented as medians (first quartile, third quartile).

FM, fermented milk protein; MP, milk protein; PL, placebo; BW, body weight.

### 3.2 Protocol compliance

There were no significant differences between the groups regarding supplementation intake compliance (FM: 100.0 [94.6, 100.0]%; MP: 98.2 [93.3,100.0]%; PL: 98.2 [95.5, 99.1]%). Moreover, no significant differences in daily soccer practice time were observed among the groups during the 8-week intervention period (FM: 75.9 [42.9, 83.9] min/d; MP: 45.7 [27.9, 78.6] min/d; PL: 64.3 [44.1, 91.6] min/d).

### 3.3 Exercise performance test

**Figure 2:**
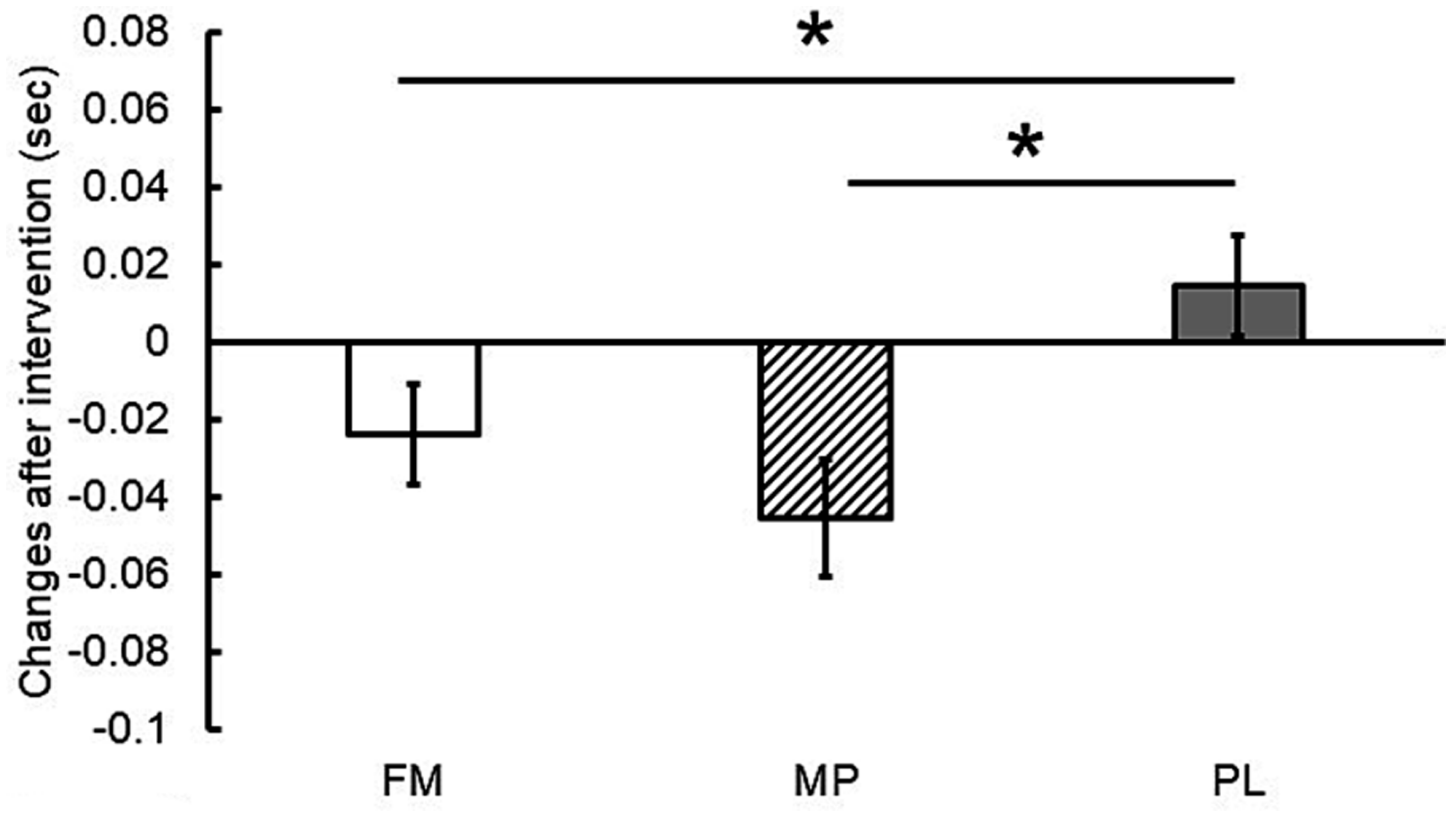
Changes in 10 m sprint time after the intervention (8 weeks). Data are presented as estimated means ± standard errors of the mean (SEM). FM, fermented milk protein; MP, milk protein; PL, placebo. *: *p* < 0.05.

**Figure 3:**
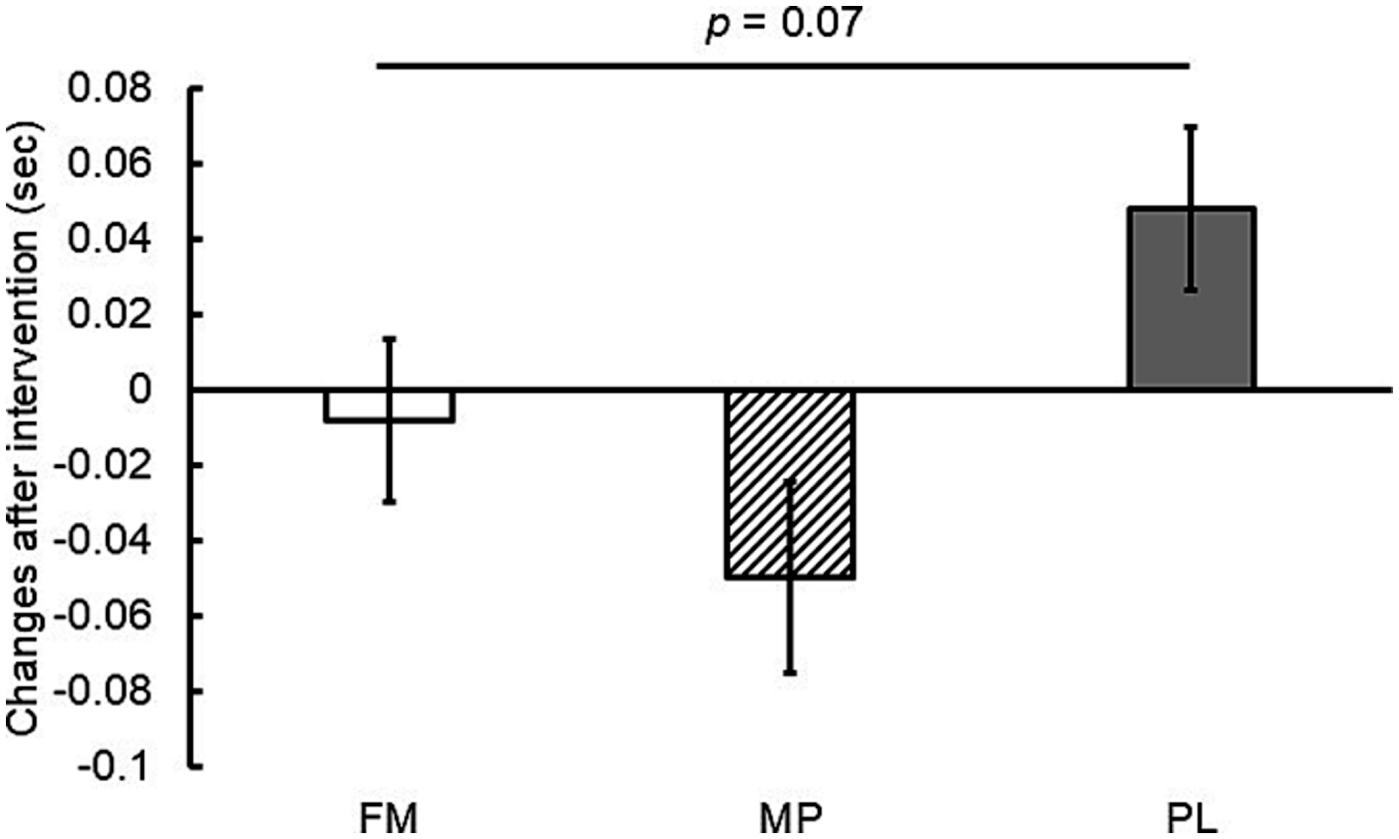
Changes in 20 m sprint time after the intervention (8 weeks). Data are presented as estimated means ± standard errors of the mean (SEM). FM, fermented milk protein; MP, milk protein; PL, placebo.

Based on the LMM, changes from baseline in the 10 m sprint time were significantly greater in the FM and MP groups than in the PL group (Figure 2). Additionally, a trend toward a greater change from baseline in the 20 m sprint time was observed in the FM group than in the PL group (*p* = 0.07) (Figure 3). No significant inter-group differences were observed for CMJ, standing jump, or YYIRT1 outcomes. Furthermore, no significant intra-group (pre- vs. post-intervention) changes were detected for these measures, and inter-group comparisons of measured values likewise showed no significant differences. These null findings are summarized in Table 3 and should be interpreted in light of the pilot sample size.

**Table 3:** Exercise performance test values pre- and post-intervention.

Variable	FM (*n* = 15)		MP (*n* = 11)		PL (*n* = 15)	
	Pre	Post	Pre	Post	Pre	Post
10 m sprint time (s)	2.11 (2.03, 2.24)	2.07 (2.00, 2.19)	2.09 (2.05, 2.20)	2.06 (2.04, 2.17)	2.17 (2.00, 2.21)	2.16 (2.06, 2.19)
20 m sprint time (s)	3.76 (3.64, 3.93)	3.73 (3.57, 3.93)	3.70 (3.61, 3.94)	3.67 (3.62, 3.89)	3.78 (3.59, 3.94)	3.81 (3.66, 3.98)
CMJ (cm)	26.86 (23.80, 29.44)	25.16 (24.28, 26.75)	26.97 (24.45, 29.74)	25.50 (24.23, 27.96)	26.97 (23.53, 29.03)	24.61 (23.37, 27.11)
Standing jump (cm)	171 (160, 178)	171 (153, 177)	167 (166, 174)	169 (164, 176)	166 (156, 178)	167 (148, 177)
YYIRT1 (m)	640 (360, 920)	520 (360, 880)	680 (460, 880)	560 (360, 840)	560 (420, 800)	520 (360, 640)

Values are presented as medians (first quartile, third quartile).

FM, fermented milk protein; MP, milk protein; PL, placebo; CMJ, countermovement jump; YYIRT1, Yo-Yo intermittent recovery test 1.

### 3.4 Body composition

**Figure 4:**
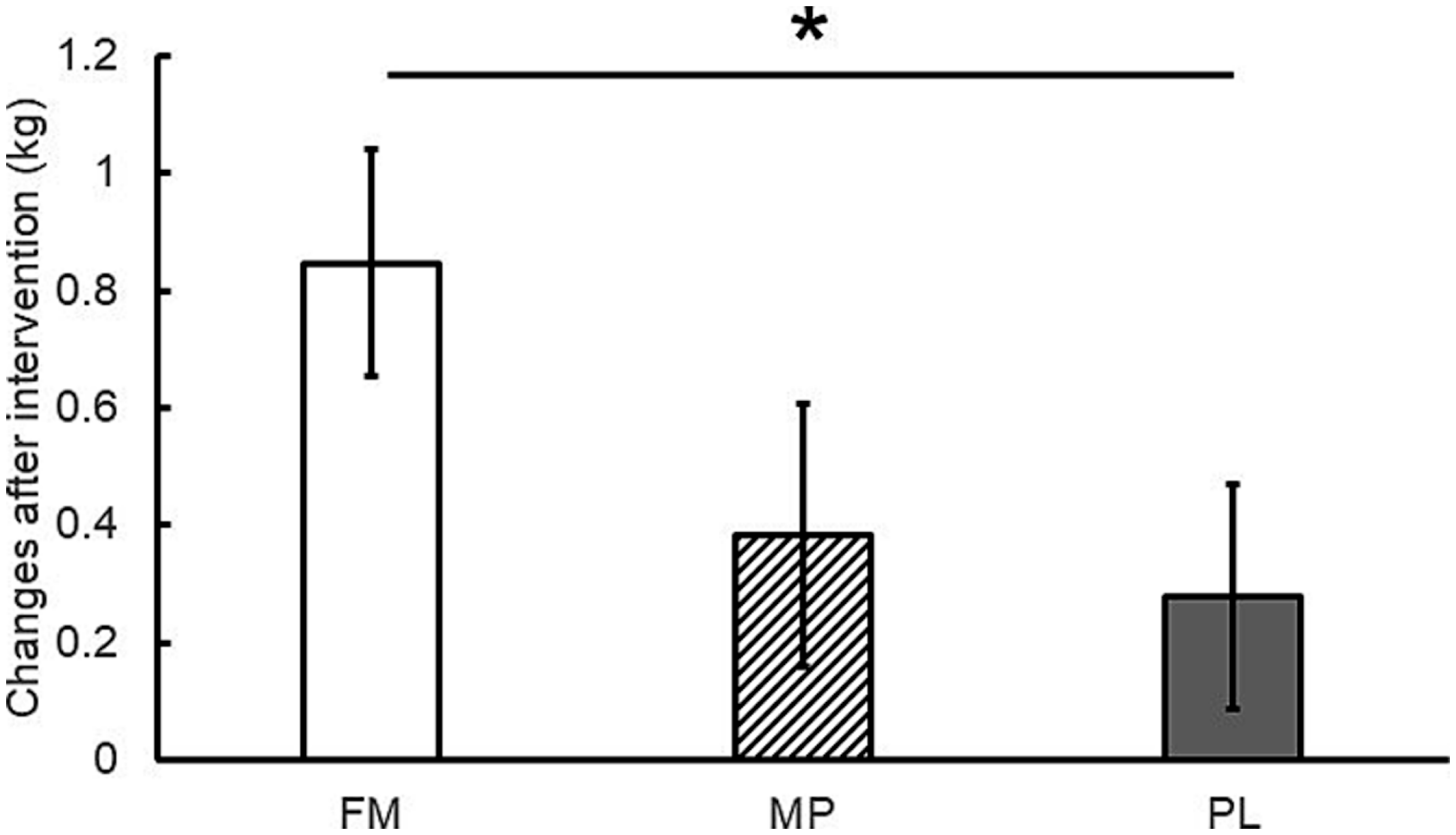
Changes in total body mass after the intervention (8 weeks). Data are presented as estimated means ± standard errors of the mean (SEM). FM, fermented milk protein; MP, milk protein; PL, placebo. *: *p* < 0.05.

Total body mass and LBM before and after the intervention are presented in Table 4. Changes in the total body mass from baseline were significantly greater in the FM group than in the PL group (Figure 4). No significant differences were observed among the groups with respect to changes in LBM. In the intra-group comparison, the total body mass of the FM group significantly increased after the intervention. No significant differences were found in the inter-group comparisons of the measured values.

**Table 4:** Body composition pre- and post-intervention.

Variable	FM (*n* = 15)		MP (*n* = 11)		PL (*n* = 15)	
	Pre	Post	Pre	Post	Pre	Post
Total body mass (kg)	36.4 (29.9, 39.7)	37.6* (30.7, 40.5)	34.4 (31.0, 36.8)	34.9 (31.3, 37.7)	34.5 (31.1, 38.6)	33.6 (31.6, 39.2)
Lean body mass (kg)	30.9 (27.1, 32.8)	29.9 (27.8, 33.6)	30.2 (27.4, 32.2)	29.6 (27.2, 32.3)	28.1 (26.4, 30.8)	28.7 (26.3, 31.1)

*p < 0.05., vs Pre. Values are presented as median (first quartile, third quartile).

FM, fermented milk protein; MP, milk protein; PL, placebo.

### 3.5 Gut microbiota analysis

In the gut microbiota analysis, a post-intervention comparison between the FM and PL groups showed that the relative abundances of *Agathobacter faecis* and *Ruminococcus bromii* were significantly higher in the FM group than in the PL group, whereas the relative abundance of *Bacteroides stercori* was significantly lower in the FM group than in the PL group (Table 5). No significant differences in the relative abundance were observed in the pre-intervention comparison between the FM and PL groups. In the post-intervention comparison between the FM and MP groups, the relative abundance of *Bacteroides stercoris* was significantly higher in the FM group than in the MP group, whereas the relative abundances of *Roseburia hominis* and *Bacteroides caccae* were significantly lower in the FM group than in the MP group (Table 6). No significant differences in relative abundance were observed in the pre-intervention comparison between the FM and MP groups. No significant differences in the relative abundance were observed in the post-intervention comparison between the MP and PL groups. In the pre-intervention comparison between the PL and MP groups, the relative abundances of *Megamonas funiformis* and *Bacteroides fragilis* were significantly lower in the PL group than in the MP group (data not shown). However, no significant differences in the relative abundance between the PL and MP groups were observed in the post-intervention comparison. In the intra-group comparison between pre- and post-intervention, the relative abundance of *Bacteroides massiliensis* was significantly increased in the FM group post-intervention (Table 7). No significant differences in the relative abundance were observed pre- and post-intervention between the MP and PL groups. No significant differences in alpha diversity were observed in the inter- and intra-group comparisons (Table 8).

**Table 5:** Gut bacteria with significant differences in relative abundance between the FM and PL groups after the intervention.

Species	Base mean	log2 fold change	Adjusted-*p* value
*Agathobacter faecis*	8.53	22.13	*p* < 0.001
*Bacteroides stercoris*	40.70	−25.07	*p* < 0.001
*Ruminococcus bromii*	30.31	9.99	*p* < 0.05.

FM, fermented milk protein; PL, placebo.

**Table 6:** Gut bacteria with significant differences in relative abundance between the FM and MP groups after the intervention.

Species	Base mean	log2 fold change	Adjusted-*p* value
*Bacteroides stercoris*	30.32	24.15	*p* < 0.001
*Roseburia hominis*	5.87	−3.43	*p* < 0.05
*Bacteroides caccae*	29.53	−7.51	*p* < 0.05

FM, fermented milk protein; MP, milk protein.

**Table 7:** Gut bacteria with significant differences in relative abundance within the fermented milk protein group between pre- and post-intervention.

Species	Base mean	log2 fold change	Adjusted-*p* value
*Bacteroides massiliensis*	11.04	22.64	*p* < 0.001

**Table 8:** Alpha diversity of the gut microbiota pre- and post-intervention.

Variable	FM (*n* = 15)		MP (*n* = 12)		PL (*n* = 15)	
	Pre	Post	Pre	Post	Pre	Post
Observed ASV	102.0 (91.0, 124.0)	125.0 (87.5, 144.5)	98.5 (80.8, 129.3)	101.0 (70.8, 121.8)	98.0 (88.0, 106.0)	111.0 (93.5, 128.0)
Chao1 Index	133.1 (126.2, 145.2)	138.8 (117.9, 168.0)	127.0 (110.7, 160.0)	120.2 (83.2, 155.7)	117.8 (98.2, 149.8)	134.3 (110.1, 161.0)
Shannon Index	2.57 (2.32, 2.93)	2.71 (2.60, 2.95)	2.54 (1.99, 2.75)	2.52 (2.23, 2.70)	2.59 (2.24, 2.74)	2.47 (2.28, 2.76)
Simpson Index	0.83 (0.81, 0.89)	0.89 (0.85, 0.90)	0.85 (0.74, 0.89)	0.85 (0.79, 0.88)	0.86 (0.80, 0.88)	0.82 (0.79, 0.88)

ASV, amplicon sequence variants.

FM, fermented milk protein; MP, milk protein; PL, placebo.

### 3.6 Association of gut microbiota with exercise performance and body composition

**Figure 5:**
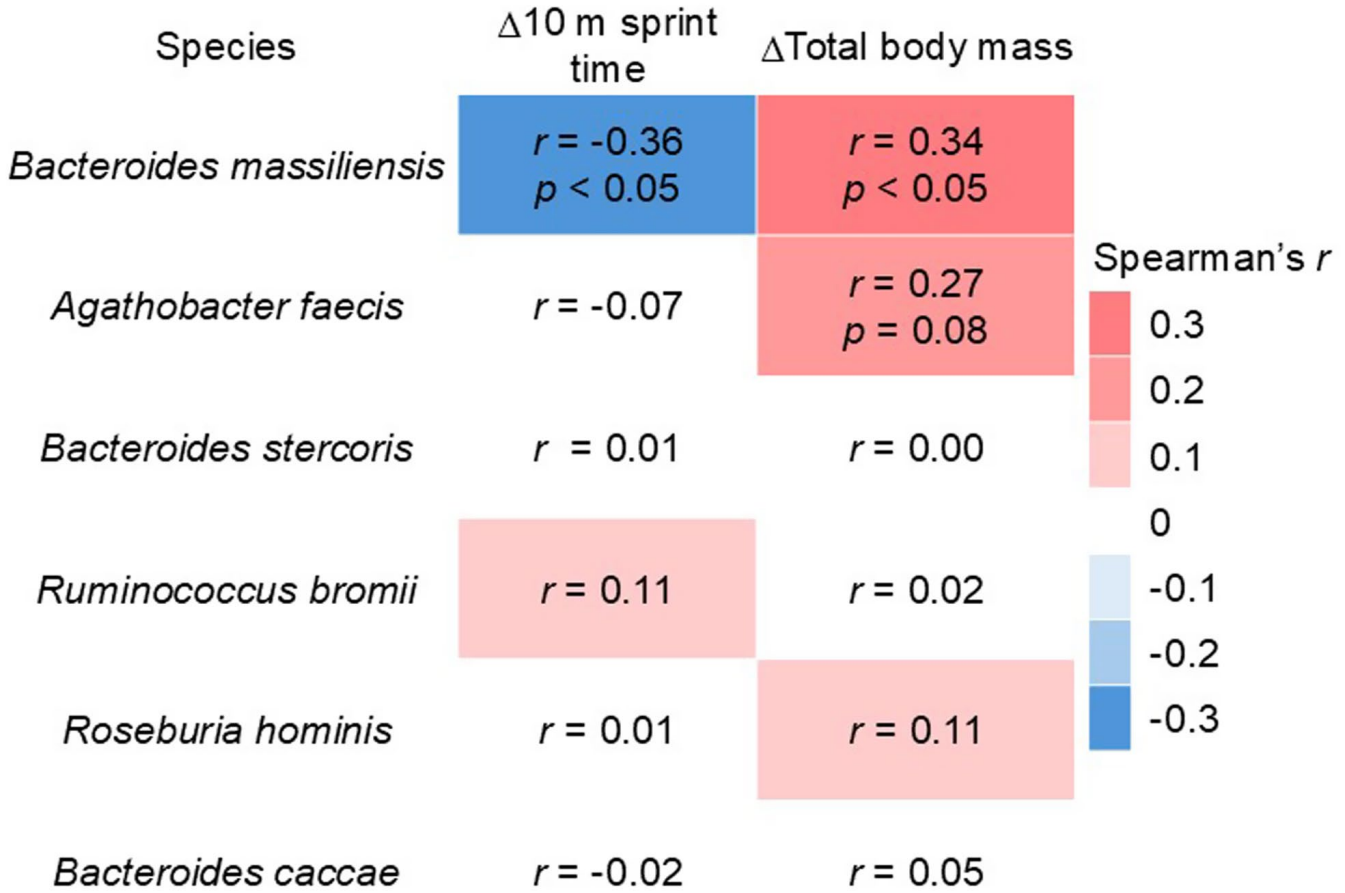
Correlation coefficients between the relative abundance of gut bacteria and 10 m sprint time and total body mass.

The correlation between the significantly different bacterial species and the change from baseline in both the 10 m sprint time and body weight was analyzed (Figure 5). The results revealed that *Bacteroides massiliensis* had a significant negative correlation with the change in the 10 m sprint time and a significant positive correlation with the change in body weight. Additionally, *Agathobacter faecis* showed a positive correlation with changes in body weight (*p* = 0.08). However, these associations are observational and do not imply causality.

### 3.7 Exploratory post-hoc stratified analysis by energy balance

**Figure 6:**
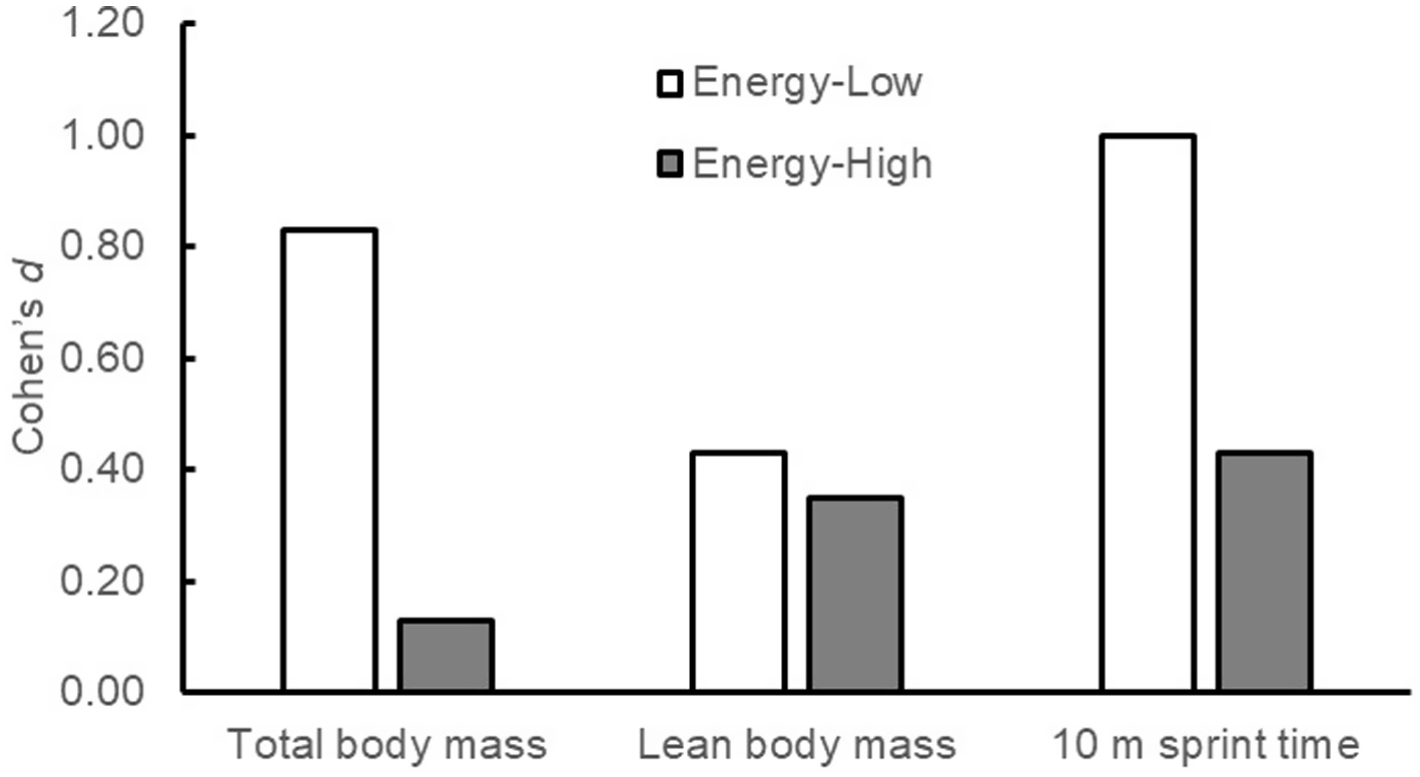
Effect sizes (Cohen’s *d*) for the changes in total body mass, lean body mass, and 10 m sprint time in the FM and PL groups, stratified by energy balance (energy-low vs. energy-high). FM, fermented milk protein; PL, placebo.

The comparison results of the FM and PL subgroups are shown in Figure 6. The stratified analysis revealed that the effect size of the fermented milk protein intervention was large for the change in body weight and 10-m sprint time (*d* = 0.83 and 1.00, respectively) in the energy-low group. For changes in LBM, the effect size was medium (*d* = 0.43) for the same group. In contrast, in the energy-high group, the effect size of the fermented milk protein intervention was either medium or small (change in body weight, *d* = 0.13; change in LBM, *d* = 0.35; change in 10 m sprint time, *d* = 0.43).

## 4 Discussion

To the best of our knowledge, this is the first randomized, double-blind, placebo-controlled trial in prepubertal young athletes that directly compares a fermented milk protein beverage with both a non-fermented milk protein beverage and an isocaloric placebo. The study integrates performance (sprint), anthropometrics (total body mass/LBM), and gut microbiota within a single protocol, and evaluates a postbiotic-containing formulation in this population. In addition, the energy-balance stratification provides hypothesis-generating insight into which children may benefit most. While preliminary by design, these features collectively comprise the principal novelty of the present work.

In this pilot randomized trial, daily fermented milk protein intake was associated with improved 10-m sprint performance and increased total body mass compared with an isocaloric placebo over 8 weeks in prepubertal boys. However, superiority over non-fermented milk protein was not consistently observed, and other performance outcomes did not differ among groups. These findings suggest potential benefits but require confirmation in adequately powered trials.

In the present study, the consumption of either fermented milk protein or milk protein beverages improved athletic performance in prepubertal children; however, no significant difference in LBM was observed among the groups. Although further research is needed to determine whether these lines of evidence apply to prepubertal children, according to previous research, four main outcome patterns have been observed in protein intervention studies combined with exercise (25). First, hypertrophy and improved performance due to protein intake. Many studies have indicated that protein or amino acid supplementation, when compared with placebo, significantly enhances an increase in muscle mass (e.g., LBM and muscle cross-sectional area) and muscle strength resulting from resistance exercise (26–32). Second, is the training effect only, with no additional protein effect on hypertrophy or performance. Several studies have shown that, while resistance training itself leads to improvements in muscle hypertrophy and athletic performance, protein consumption provides no additional benefits compared with placebo (33–40). Factors contributing to this may include the participants already consuming sufficient protein from their habitual diet before the intervention, which may dilute or mask the additive effects of supplementation (33, 34). Additionally, a relatively short intervention period, such as 8 weeks, may be dominated by initial training adaptations (particularly neural adaptations), which may not be sufficient to detect subtle effects of supplementation (25). Third, hypertrophy without performance improvement. Some reports indicated that while an increase in muscle mass was observed in the protein supplementation group, there was no significant difference in strength gain compared to the placebo group (41–43). Fourth, performance improvement without hypertrophy. Although few studies have reported this, there are cases where protein supplementation does not provide an additional effect on muscle mass but still leads to improved strength or specific aspects of athletic performance (44, 45), which is consistent with the findings of the current study. Similar results have been documented in clinical trials involving adults. However, to the best of our knowledge, this is the first study to report that protein supplementation improves athletic performance in prepubertal children. Improvements in strength and power are not solely dependent on muscle hypertrophy. Mechanistically, improvements in sprint performance without detectable LBM change could possibly reflect early neural adaptations and recovery support rather than hypertrophy (46); this interpretation remains speculative. Prior studies investigating resistance training interventions in prepubertal children have reported that resistance training resulted in muscle strength gains primarily due to neural adaptations but no corresponding increase in muscle mass (47). Protein consumption may facilitate earlier improvements in strength by enabling adaptation to higher training intensity and volume and by supporting recovery from resistance training (48). Regarding neural adaptations, research has been reported on the Milk Fat Globule Membrane (MFGM), a component of milk. Studies in healthy young adults have shown that consuming the MFGM in combination with exercise training is effective in improving explosive physical capabilities, particularly muscle strength and power (49, 50). Both the fermented milk protein and milk protein beverages used in this study contained whey protein concentrate as the primary ingredient, which is known to contain a small amount of MFGM. Further investigation of the combined effects of proteins and MFGM is warranted.

**Figure 7:**
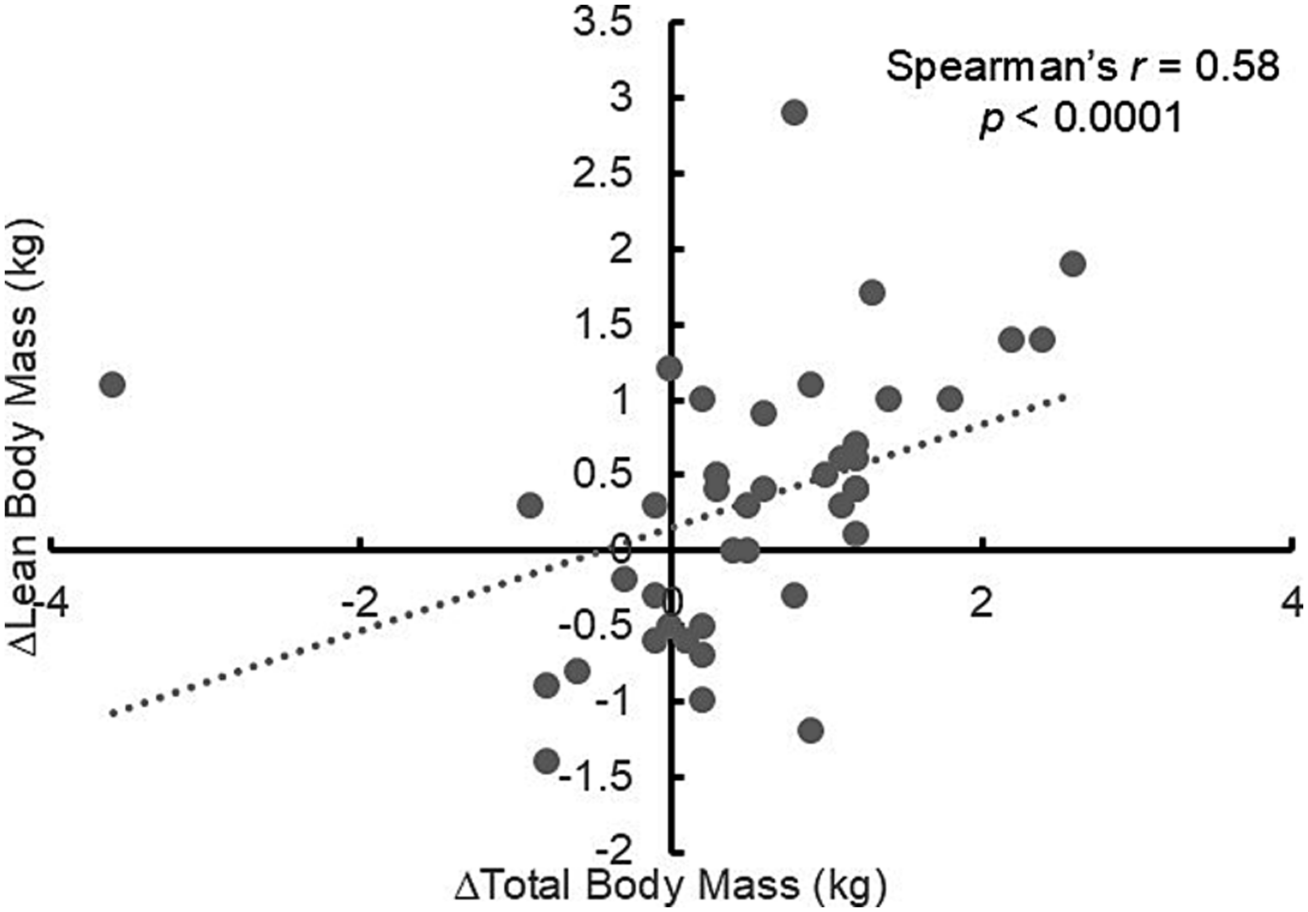
Correlation between the change in total body mass and change in lean body mass.

In this study, the group that consumed fermented milk protein beverages showed an increase in total body mass. Animal studies have reported that fermented milk promotes greater weight gain than unfermented milk (14, 15). The proposed mechanism involves the potential for improved digestibility and bioavailability of fermented milk proteins (7). The dairy proteins in fermented milk are altered by the fermentation process, making them easier to digest than those in unfermented milk. Furthermore, fermented milk proteins form a soft, acidic gel that readily mixes with stomach acid, making it less prone to coagulation. This facilitates predigestion by pepsin and enables easier transit to the small intestine. Consequently, the rate of amino acid absorption is expected to increase, thereby boosting MPS. Previous research has demonstrated that following fermented milk consumption, blood TAA concentrations are higher, the time to reach peak concentration is shorter (51), and MPS is elevated compared with unfermented milk intake (8, 9). In growing children, weight gain is associated with increased LBM. For instance, Kida *et al.* showed that changes in body weight and LBM were nearly identical when 11-year-old boys were tracked for over 1 year (52). Consistent with this, our analysis revealed a significant correlation between changes in total body mass and LBM (Figure 7). To the best of our knowledge, this is the first study to investigate the effects of fermented milk proteins on the physique of prepubescent children. Further large-scale studies are required to further validate our findings.

For normal child development, appropriate intake of energy and essential amino acids, in addition to proteins, is necessary (10, 11). We conducted a stratified analysis focusing on the balance between energy intake and expenditure. These results suggest that fermented protein intervention is particularly effective in children with an insufficient energy intake. Given that these results were based on a limited sample size, further research is warranted regarding the effects of fermented milk protein consumption on energy intake status.

Recently, postbiotics have gained attention as a new class of antibiotics. According to a consensus definition, a postbiotic is defined as a ‘preparation of inanimate microorganisms and/or their components that confers a health benefit on the host’ (53). The fermented milk protein beverage used in this study is a postbiotic, containing 2.6 × 10^11^ CFU of inanimate lactic acid bacteria. It has been suggested that the mechanism of action for the health benefits conferred by postbiotics may be similar to that of probiotics (54, 55). Several studies have reported on the consumption of bacterial cells as probiotics and their relationship with muscle mass and strength. A meta-analysis by Prokopidis et al. showed that probiotic supplementation enhanced both muscle mass and overall muscle strength compared with a placebo (56). However, no beneficial effects were observed for total LBM. Furthermore, the stratified analysis suggested that probiotics may have a more pronounced effect on muscle mass gain in populations under 50 years of age. This is the first study to evaluate the effects of a postbiotic intervention on exercise performance, total body mass, and LBM in prepubertal children. Although we observed no significant group differences in LBM, a group difference was found in total body mass, suggesting that further research is required. One proposed mechanism of action of postbiotics is the modulation of resident microbiota (53). Postbiotics, through their inanimate components, are thought to play a crucial role in the gut microbiota, even if only in the short term. In this study, consumption of a fermented milk protein beverage increased *Bacteroides massiliensis*, and a correlation was observed between its relative abundance and changes in both exercise performance and body weight. A study by Chen *et al.* among 5-year-old children demonstrated a positive correlation between the amplicon sequence variants of *Bacteroides massiliensis* and total lean soft tissue mass; however, this correlation was not statistically significant (57). Conversely, associations have been shown between children’s anthropometric indices such as total mass, lean soft tissue mass, total fat mass, and trunk fat mass and microbial alpha diversity indices (57). In the current study, no significant differences were observed between the groups regarding alpha diversity indices. At post-intervention, the FM group tended to have higher alpha diversity than the PL group according to several indices (Shannon Index, *p* = 0.087; Simpson Index, *p* = 0.056). Further investigation is required regarding the relationship between the gut microbiota, children’s anthropometrics, and exercise performance.

Despite these important findings, the present study is subject to several limitations that should be acknowledged. First, the relatively small sample size (*n* = 44) restricted the statistical power and increased the risk of type II error. Second, the short 8-week intervention period prevents us from drawing conclusions regarding the long-term sustainability of the intervention effects. Third, a constraint relates to our dietary assessment methodology. Nutritional intake estimated using the BDHQ15y is subject to inherent measurement error and reporting bias compared with detailed methods like the weighed food record. Moreover, this method may not fully capture changes in dietary protein from non-study foods during the intervention. This limitation is considered in the interpretation. Furthermore, although all beverages were identically packaged and matched for pH, minor differences in taste or mouthfeel between fermented and non-fermented formulations could not be formally excluded. We did not conduct a sensory discrimination test; therefore, blinding may not have been fully effective, which we acknowledge as a limitation. In addition, body composition was assessed at a fixed evening time to reduce diurnal variation; however, fluid intake could not be strictly controlled in free-living children. Given the hydration sensitivity of BIA, LBM estimates should be interpreted with caution. Finally, the convenience sampling approach may have introduced a volunteer bias since participants are likely more motivated than the general population, thereby limiting the external validity of the results. Taken together, future research should address these limitations by incorporating a larger, randomized sample, a longer follow-up period, and more rigorous dietary assessment methods to confirm the present findings.

In this pilot randomized trial, we found that consumption of a fermented milk protein beverage was associated with improved 10-m sprint performance and increased total body mass compared with an isocaloric placebo in prepubertal boys who play soccer. However, superiority over non-fermented milk protein was not consistently demonstrated, and other performance outcomes did not differ among groups. Larger and longer trials are warranted to confirm our findings and clarify underlying mechanisms.

## Data Availability

The raw sequence data generated in this study have been deposited in the DNA Databank of Japan (DDBJ) Sequence Read Archive under the BioProject numbers PRJDB40612.
